# The role of the globular heads of the C1q receptor in HPV-16 E2-induced human cervical squamous carcinoma cell apoptosis via a mitochondria-dependent pathway

**DOI:** 10.1186/s12967-014-0286-y

**Published:** 2014-10-05

**Authors:** Zheng-lin Chen, Ya-juan Su, Hui-lin Zhang, Ping-qing Gu, Ling-juan Gao

**Affiliations:** Clinical Laboratory, Jiangsu Provincial Official Hospital, Nanjing, 210024 China; Clinical Laboratory, Harbin Medical University Cancer Hospital, Harbin, 150080 China; State Key Laboratory of Reproductive Medicine, Department of Clinical Laboratory, Nanjing Maternity and Child Health Care Hospital Affiliated to Nanjing Medical University, Nanjing, 210004 China; Clinical Laboratory, Nanjing Maternity and Child Health Care Hospital, Tianfei Alley, Nanjing Mochou Road, 210004 Nanjing, P.R. China

**Keywords:** Human papillomavirus type 16 (HPV-16) E2, Receptor for the globular heads of the human C1q (gC1qR), Mitochondrial dysfunction, Apoptosis, Human cervical squamous carcinoma cells

## Abstract

**Background:**

Human papillomavirus type-16 (HPV-16) E2 protein acts as a transcriptional modulator and plays a key role in regulating many biological responses. The purpose of this study was to investigate the relationship between HPV-16 E2, the receptor for the globular heads of human C1q (gC1qR) gene expression, mitochondrial dysfunction and apoptosis regulation in human cervical squamous carcinoma cells (C33a and SiHa).

**Methods:**

HPV-16 E2 and gC1qR expression was examined using real-time PCR and western blot analysis. Apoptosis in C33a and SiHa cells was assessed by flow cytometry. Mitochondrial function was detected via ROS generation, the amount of cytosolic Ca^2+^, and changes in the mitochondrial membrane potential (Δψm).

**Results:**

The expression of the HPV-16 E2 and gC1qR gene significantly decreased in human cervical squamous carcinoma samples relative to the non-cancerous cervix samples. C33a and SiHa cells that were transfected with a vector encoding HPV-16 E2 displayed significantly increased gC1qR gene expression and mitochondrial dysfunction as well as an up-regulation of cellular apoptosis, which was abrogated by the addition of gC1qR small-interfering RNA (siRNA).

**Conclusions:**

These data support a mechanism whereby gC1qR plays an important role in HPV-16 E2-induced human cervical squamous carcinoma cell apoptosis via a mitochondria-dependent pathway.

**Electronic supplementary material:**

The online version of this article (doi:10.1186/s12967-014-0286-y) contains supplementary material, which is available to authorized users.

## Background

Cervical cancer is the second most frequent malignancy in women and is responsible for substantial number of morbidities and mortalities throughout the word [1]. Molecular and epidemiological studies have shown that infection with a high-risk human papillomavirus (HPV) type is a major factor in the development of cervical cancer and is responsible for nearly all cases of cervical cancer [2]. Of the over 100 HPV types known thus far, HPV-16 is the most common high-risk virus and causes more than 50% of all cases of cervical carcinoma [3]. During carcinogenesis, HPV-16 DNA integrates into the host cell genome, resulting in a loss of expression of the regulatory gene E2, which is relatively conserved among papillomaviruses [4]. In addition to being a transcriptional regulator, the E2 protein negatively regulates the HPV viral oncogenes E6 and E7 in benign lesions [5]. E2 proteins from high-risk HPV have been proven to affect cellular processes such as anti-proliferation, apoptosis, regulation of the viral life cycle, and gene expression [6,7]. Previous studies have shown a direct relationship between HPV E2 gene expression and mitochondrial dysfunction in apoptosis in cervical cancer cells [8].

The main function of mitochondria is to produce energy by synthesising ATP. Reactive oxygen species (ROS) production contributes to mitochondrial damage in the pathological level and also plays an important role in redox signalling from the organelle to the rest of the cell [9]. Studies have demonstrated that the high-risk HPV-16 E2 protein can induce mitochondrial dysfunction by regulating protein expression and the localisation of proteins to mitochondrial membranes [10]. The receptor for the globular head of C1q, gC1qR, was initially identified as a protein within the mitochondrial matrix [11] that could mediate many biological responses, including growth perturbations, morphological abnormalities and the initiation of apoptosis [12]. This study aimed to comprehensively identify the effect of the gC1qR gene on HPV-16 E2-induced apoptosis of cells and to investigate whether the gC1qR-induced biological changes acted through a mitochondria-dependent pathway in HPV-16 E2-transfected cervical squamous carcinoma cells.

## Materials and methods

### Chemicals and reagents

The human cervical squamous carcinoma cell lines C33a (HPV-16 negative) and SiHa (HPV-16 positive) were purchased from Hangzhou Hibio Bio-tech Co., Ltd (Hangzhou, Zhejiang, China). Dulbecco’s Modified Eagle’s Medium (DMEM) powder, penicillin and streptomycin were purchased from Invitrogen/Gibco (Grand Island, NY, USA). The Phototope-HRP Western Blot Detection System, including an anti-mouse IgG, an HRP-linked antibody, a biotinylated protein ladder, 20X LumiGLO Reagent and 20X peroxide, was purchased from Cell Signalling Technology (Beverly, MA, USA). Lipofectamine 2000, was purchased from Invitrogen (Carlsbad, CA, USA). 2’ ,7’-dichlorodihydrofluorescein diacetate (H_2_DCFDA) was obtained from Molecular Probes. The Annexin V-FITC/Propidium Iodide (PI) Flow Cytometry Assay Kit was purchased from Invitrogen (Carlsbad, CA, USA). Antibodies targeting HPV-16 E2, gC1qR, and actin were purchased from Santa Cruz (Santa Cruz, CA, USA) and Cell Signalling Technology. The pcDNA-HPV-16 E2 and pcDNA-HPV-16 E2 mutant (mut) plasmids were kindly supplied by Hangzhou Hibio Bio-tech Co., Ltd. gC1qR small-interfering RNA (siRNA) and negative siRNA (siRNA directed toward an unrelated gene as a negative control) were synthesised by Wuhan Genesil Biotechnology Co., Ltd (Wuhan, China). Cell culture supplies were purchased from Life Technologies (Gaithersburg, MD, USA). Unless otherwise specified, all of the other reagents were of analytical grade.

### Tissue procurement and preparation

Between October 2009 and January 2012, we recruited women who underwent radical hysterectomies due to cervical carcinoma at Nanjing Maternity and Child Health Care Hospital. This study was approved by the Ethical Committee of the Chinese Academy of Sciences and the Nanjing Maternity and Child Health Care Hospital in Nanjing. All of the study participants gave informed consent for the collection of the tissues and blood samples. Human cervical cancer specimens were obtained from 30 HPV-16-positive patients (median age of 45 years, age range between 22–59 years), which were studied along with a control group (median age of 43 years, age range between 21-54 years). Some cervix tissues from non-cervical cancer patients who have had a hysterectomy for hysteromyoma or adenomyosis etc were collected, the other from patients who have had a tissue biopsy for non cancer diagnoses. From those tissues, thirty cases from the patients (HPV-16 is positive, and the HPV typing was detected using gene chip technique in this study) were chosen as the control group, which pathological diagnosis was mild cervicitis or have no obvious pathological changes. The human cervical squamous cell carcinoma tissues and non-cancerous cervix tissues were all reviewed by a pathologist and histologically. The infection of other sexually transmitted pathogens including CT, NG, GV, MG, TV, MH, and HSV-2 was detected by routine clinical microbiology methods before the HPV analysis.

### Cell culture

The human cervical carcinoma cell lines C33a and SiHa were cultured in DMEM medium containing 10% foetal bovine serum, 1% nonessential amino acids, 2 mM glutamine, and antibiotics (100 units/ml penicillin and streptomycin). The cells were maintained in a humidified 5% CO_2_ incubator at 37°C.

### Cloning and transfection of the HPV-16 E2 plasmids

The full-length HPV-16 E2 open reading frame (ORF) was constructed in-frame into the pcDNA 3.1 expression plasmid (Invitrogen, Carlsbad, CA) by PCR amplification using the BamHI and EcoRI restriction sites according to the pBR322 reference clone. Primer-F (5′-GAT GGA GAC TCT TTG CCA ACG-3′) and Primer-R (5′-TCA TAT AGA CAT AAA TCC AGT AGA C-3′) were used to clone the HPV-16 E2. The mutant HPV-16 E2 plasmid was created by PCR mutagenesis using the Primer-F (5′-GAT GGA GAC TCT TTG CCA ACG-3′) and mutant Primer-R (5′-TCC CAT TCT CTG GCC TTG TAA ATA GCA CA ***TGC*** TAG-3′), where the mutated codons are denoted in bold and italic. The HPV-16 E2 mutant reduced DNA replication activity and transactivation regulation [13]. The resulting pcDNA-HPV-16 E2 vector and mutant HPV-16 E2 vector were then transfected into C33a and SiHa cells, respectively. Twenty-four hours after plating, the cells were serum starved in RPMI-1640 medium containing 0.5% FBS for an additional 24 h until the cells became quiescent. Following serum starvation, pcDNA-HPV-16 E2 was transfected into the cells (90% confluent) at passage numbers 6, 9 and 12 using Lipofectamine™ reagent (Life Technologies, Inc.) according to the manufacturer’s protocol. Reporter gene levels were normalised to the amount of total protein, and each experiment was independently performed three to five times.

### gC1qR siRNA-expressing plasmid construction

To silence the objective genes, the siRNA target gene sequence was designed to be homologous to nucleotides 408-426 of the human gC1qR mRNA. The forward siRNA sequence was 5′-AAC AAC AGC AUC CCA CCA ACA UU-3′. The 5′ end oligonucleotides contained BamHI and HindIII restriction site overhangs. The gC1qR siRNA-expressing plasmid was constructed using pGenesil-1 as the vector backbone. The siRNA was synthesised, annealed and ligated into the BamHI and HindIII restriction sites in the linearised pGenesil-1 expression vector. At the same time, a vector containing the siRNA for an unrelated gene was used as a negative control.

### Scanning and transmission electron microscopy

Biopsies were taken immediately after surgery. Tumour specimens were obtained by cutting longitudinal sections of 3-5-mm maximum thickness, which were immersed in phosphate-buffered 2.5% glutaraldehyde for 2 h. Following an overnight washing with 0.1 M sodium phosphate buffer, the tissue blocks were post-fixed in 1% OsO_4_ in a 0.1 M phosphate buffer (pH 7.4) for 1 h, stained with 1% uranyl acetate, and then dehydrated in an acetone gradient. For transmission electron microscopy, ultrathin (60-70 nm) sections were stained with uranyl acetate and lead citrate. The cell morphology was examined at 3700X and 12500X magnification and photographed using a JEOL JEM-2000EX transmission electron microscope (Tokyo, Japan).

### Western blot analysis

Following various treatments for 48 h, cells were harvested in ice-cold PBS, pelleted at 15,000 rpm for 5 min, and then incubated in lysis buffer containing 50 mM Tris-HCl (pH 7.4), 0.5% NP-40, 150 mM NaCl, 50 mM NaF, 1 mM Na_3_VO_4_, 1% Triton X-100, 1 mM EDTA, 1 mM PMSF, 10% glycerol, and protease inhibitor cocktail on ice for 30 min. The supernatants were centrifuged for 20 min at 13,000 rpm at 4°C. The protein was estimated using the Bradford reagent. Equal amounts of protein were loaded and separated on a 10-15% SDS-polyacrylamide gel and then transferred onto a PVDF membrane. The membranes were blocked for 1 h in 5% non-fat milk in PBST (PBS containing 0.05% Tween 20) and then incubated with the appropriate primary antibodies against HPV-16 E2 or actin at a 1:500 dilution. The membrane was washed in PBST and incubated with the secondary IgG HRP-conjugated antibody at a 1:500 dilution. The protein bands were visualised using the enhanced chemiluminescence (ECL) Western Detection System, and the densitometry analysis was performed on the scanned immunoblot images using the Image J gel analysis tool.

### Assay of intracellular ROS

ROS production was measured using the cell-permeable probe H_2_DCFDA, which preferentially measures peroxides. Briefly, C33a and SiHa cells were grown on cover slips and incubated with 10 μM H_2_DCFDA under various conditions for 15 min in the dark. The cells were then lysed with RIPA buffer in ice-cold conditions [14]. H_2_DCFDA fluorescence was detected using fluorescence microscopy at an excitation wavelength of 488 nm and an emission wavelength of 530 nm. A spectrofluorometer with a slit width of 5 nm was used to quantify the fluorescence levels of the supernatants. The experiments were repeated at least 10 times. The results were determined according to the increase in fluorescence intensity with respect to normoxic untreated controls by subtracting the basal fluorescence levels.

### Measurement of the intracellular Ca^2+^ concentration ([Ca^2+^]i)

Fluo-4 AM fluorescence was used to quantify the intracellular Ca^2+^ levels. C33a and SiHa cells were treated under various conditions at the indicated times and then washed with ice-cold PBS. The cells were resuspended in 1 mL of PBS and incubated with 5 mL of 1 mM Fluo-4 AM for 1 h. The fluorescence intensity was detected using a Beckman Coulter Paradigm™ Detection Platform at an excitation wavelength of 485 nm and an emission wavelength of 530 nm to determine the intracellular Ca^2+^ concentrations. Fluorometric measurements were performed in 10 different sets and expressed as the fold increase in fluorescence per microgram of protein compared with the control group.

### Measurement of the mitochondrial membrane potential (Δψm)

The loss of mitochondrial membrane potential (Δψm) was measured in C33a and SiHa cells after treatment under varying conditions at different time intervals using the fluorescent cationic dye JC-1, which is a mitochondria-specific fluorescent dye [15]. The dye accumulates in mitochondria with increasing Δψm under monomeric conditions and can be detected at an excitation wavelength of 485 nm and an emission wavelength of 530 nm. C33a and SiHa cells that had undergone the various treatments were washed with serum-free medium after 60 h of growth and incubated with 10 μM JC-1 at 37°C. Then, the C33a and SiHa cells were resuspended in medium containing 10% serum, and the fluorescence levels were measured at the two different wavelengths. The data are representative of 10 individual experiments.

### Detection of apoptotic cells

Apoptosis measurements were performed using Annexin V-FITC/propidium iodide staining with flow cytometry analysis. After different treatments at the indicated times, C33a and SiHa cells were washed and resuspended in binding buffer (2.5 mM CaCl_2_, 10 mM HEPES, pH 7.4, and 140 mM NaCl) before being transferred to a 5-mL tube. The cells were incubated in the dark with 5 μL each of Annexin V-FITC and propidium iodide for 15 min. The binding buffer was then added to each tube, and the samples were analysed using a Beckman Coulter Epics XL flow cytometer. The normal cells were found in the Q1_LL region, and the early and late apoptotic cells were distributed in the Q1_LR and Q1_UR regions, respectively. The necrotic cells were located in the Q1_UL region.

### Statistical analysis

Unless otherwise indicated, the results represent the mean ± standard deviation (SD). Differences between the various datasets were tested for significance using Student’s *t*-test, and *p*-values less than 0.05 were considered significant (*p < 0.05; **p < 0.01; ^#^*p* > 0.05).

## Results

### The expression of the HPV-16 E2 and gC1qR gene in human cervical tissue

**Figure 1:**
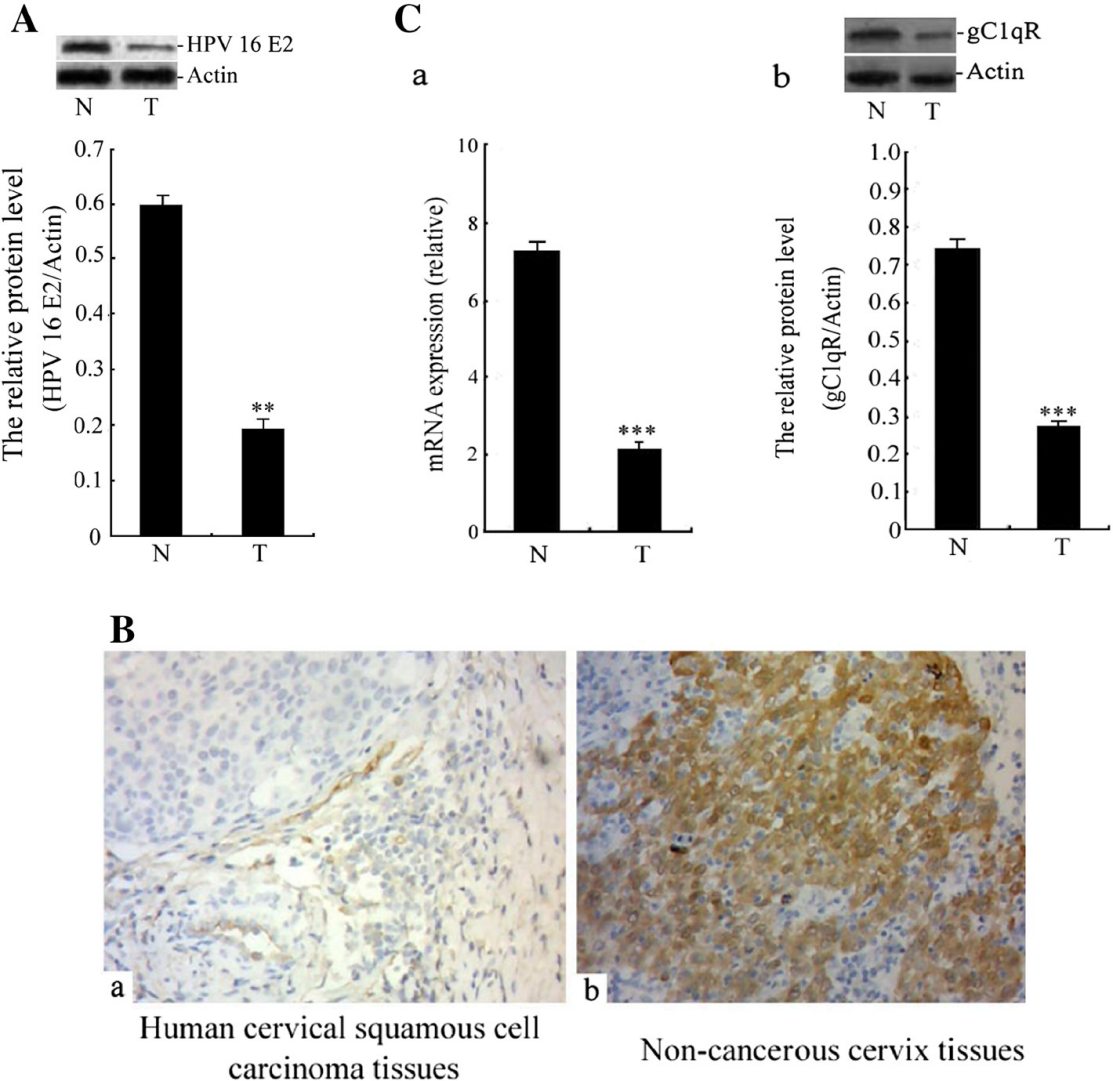
**The level of HPV-16 E2 and gC1qR protein in human cervical tissue. A**: Relative HPV-16 E2 protein levels are shown between human cervical squamous cell carcinoma tissues (T) and non-cancerous cervix tissues from patients who have had a hysterectomy for hysteromyoma or adenomyosis etc (N). The expression of the HPV-16 E2 protein was measured using western blot analysis. The graph shows the relative HPV-16 E2 protein levels, which were quantified and normalised to β-actin. The results shown are the mean ± SD of 30 independent experiments (n = 30), each conducted in triplicate. Student^’^s *t*-test: T versus N. **p < 0.01. **B**: The gC1qR levels in human cervical tissue. The results of immunohistochemical staining. The positive results for the gC1qR antigen in human cervical tissues by immunohistochemistry (200X). **a**: Staining of the monoclonal anti-gC1qR antibody in human cervical squamous cell carcinoma tissues. **b**: Staining of the monoclonal anti-gC1qR antibody in non-cancerous cervix tissues. **C**: Relative gC1qR expression levels are shown between human cervical squamous cell carcinoma tissues (T) and non-cancerous cervix tissues (N). **a**: The mRNA expression level of gC1qR was analysed by real-time PCR. **b**: The expression of the gC1qR protein was measured using western blot analysis. The graph displays the relative gC1qR protein levels normalised to β-actin. The results shown are the mean ± SD of 30 independent experiments (n = 30), each conducted in triplicate. Student’s *t*-test: N versus T. ***p < 0.001.

To investigate the relationship between the expression of HPV-16 E2, gC1qR and human cervical squamous cell carcinoma, HPV-16 E2 and gC1qR expression levels were analysed in 30 cases of human cervical squamous cell carcinoma and 30 cases of non-cancerous cervix tissues. The HPV-16 E2 protein expression was analysed by western blot analysis (Figure 1A). The results showed that the expression levels of HPV-16 E2 protein were significantly decreased in human cervical squamous cell carcinoma tissues (T) compared with non-cancerous cervix tissues (N). Meanwhile, our results further analyzed the gC1qR expression by immunohistochemistry, real-time PCR and western blot analysis in human cervical tissues. As is shown in Figure 1B-C, the gC1qR expression significantly decreased in human cervical squamous cell carcinoma tissue (T) when compared with non-cancerous cervix tissues (N). These findings suggested that HPV-16 E2 and gC1qR gene might play a negative role in the survival of human cervical squamous cell carcinomas.

### Forced expression of HPV-16 E2 induces apoptosis in C33a and SiHa cells

**Figure 2:**
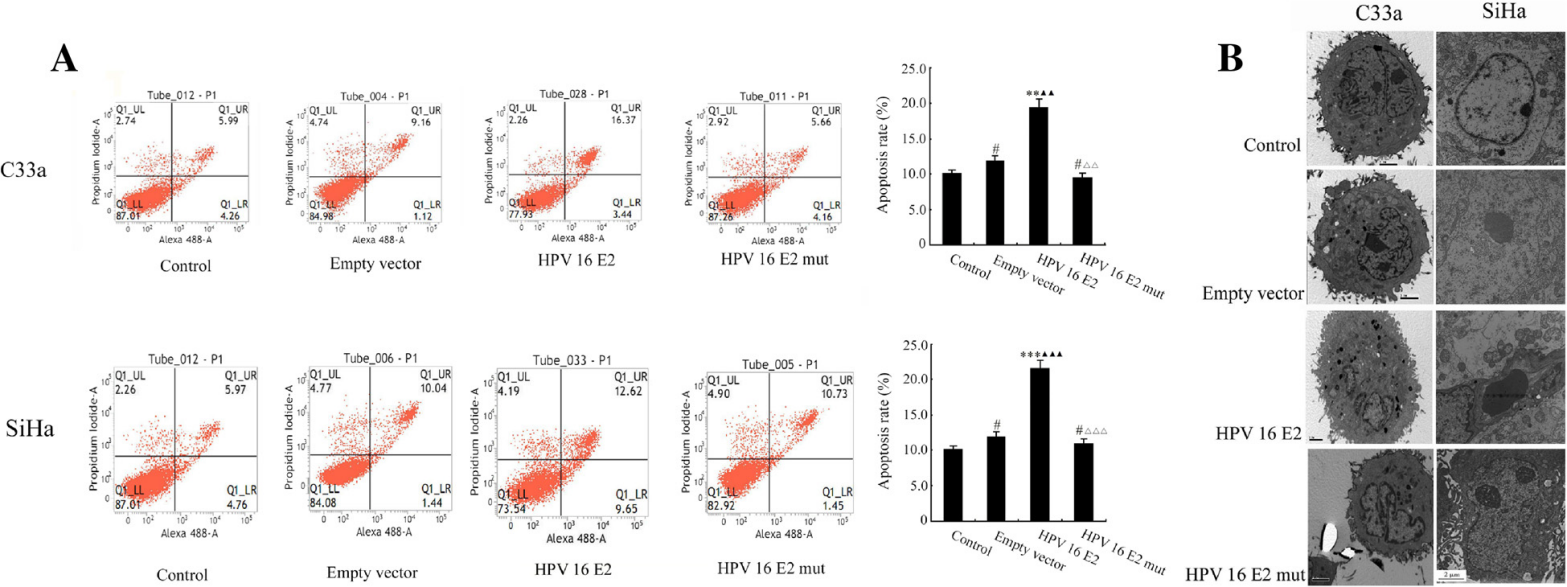
**Effects of HPV-16 E2 on apoptosis in C33a and SiHa cells. A:** The apoptotic death of cells. C33a and SiHa cells were transfected with the empty vector, the HPV-16 E2 vector, or the HPV-16 E2 mutant vector. At 48 h post-transfection, apoptosis was assessed by flow cytometry analysis to detect the subG1 populations. **p < 0.01, ***p < 0.001, ^#^ *p* > 0.05 versus the plain medium group (control); ^▲▲^ *p* < 0.01, ^▲▲▲^ *p* < 0.001 versus the empty vector group; ^△△^ *p* < 0.01, ^△△△^ *p* < 0.001 versus the HPV-16 E2 group. **B:** The morphology of the C33a and SiHa cells observed with an electron microscope. No morphologic changes were observed in cells treated with plain medium (control), empty vector, or HPV-16 E2 mutant vector (3700X). Apoptotic bodies were observed in HPV-16 E2 vector-transfected cells (3700X).

To determine if the accumulation of HPV-16 E2 could trigger apoptotic death, C33a and SiHa cells were double-stained with Annexin V and PI and assessed by flow cytometry following treatment with the empty vector, the HPV-16 E2 vector, or the HPV-16 E2 mutant vector for 48 h. The early and late apoptotic cells were distributed in the Q1_LR and Q1_UR regions, respectively. The necrotic cells were located in the Q1_UL region. Figure 2A shows that the accumulation of the HPV-16 E2 protein increased the number of C33a and SiHa cells in the Q1_LR and Q1_UR regions in the HPV-16 E2-transfected group. In contrast, there was no change in the Q1_LR and Q1_UR regions of in the empty vector or HPV-16 E2 mutant group compared with the unmodified media group (control). The number of cells in the Q1_LR and Q1_UR regions was notably decreased in the cells transfected with the HPV-16 E2 mutant vector than when the HPV-16 E2 vector was used. Visualisation of the HPV-16 E2 vector-transfected cells at 48 h with an electron microscope (Figure 2B) showed characteristic pathological subcellular changes early on during the chromatin-condensation phase. These changes included electron-dense nuclear material that was aggregated peripherally under the nuclear membrane and apoptosis bodies consisting of the cytoplasm with tightly packed organelles. However, in the plain medium (control) group, empty vector group, and HPV-16 E2 mutant vector group, the morphology of the C33a and SiHa cells showed no obvious apoptotic features.

### The effect of gC1qR on HPV-16 E2-induced mitochondrial dysfunction in C33a and SiHa cells

In previous experiments, our results revealed that the forced expression of gC1qR induced mitochondrial dysfunction (including the production of ROS, cellular calcium ion influx, and decrease of mitochondrial membrane potential) in cervical squamous carcinoma cell lines (Additional file 1: Figure S1), these findings suggested that the gC1qR play a negative role in the survival of cervical cancer cells. To more completely understand the role of gC1qR on HPV 16 E2-induced mitochondrial dysfunction and apoptosis in cervical squamous carcinoma cell lines. Firstly, the HPV 16 E2-induced gC1qR expression levels were measured via real-time PCR and western blot analysis (Additional file 2: Figure S2). This finding suggests that HPV 16 E2 could induce the expression of gC1qR gene.

Secondly, C33a and SiHa cells were treated with plain medium (control), negative siRNA vector and gC1qR siRNA vector for 48 h. The expression of gC1qR was detected to observe the silence of gC1qR gene (Additional file 3: Figure S3). Lastly, C33a and SiHa cells were treated with plain medium, HPV 16 E2 vector, HPV 16 E2 + gC1qR siRNA vector and HPV 16 E2 + negative siRNA vector for the indicated time periods. The expression of gC1qR was detected again (Additional file 4: Figure S4), these dada indicated that gC1qR siRNA could effectively silence the target gC1qR gene.

**Figure 3:**
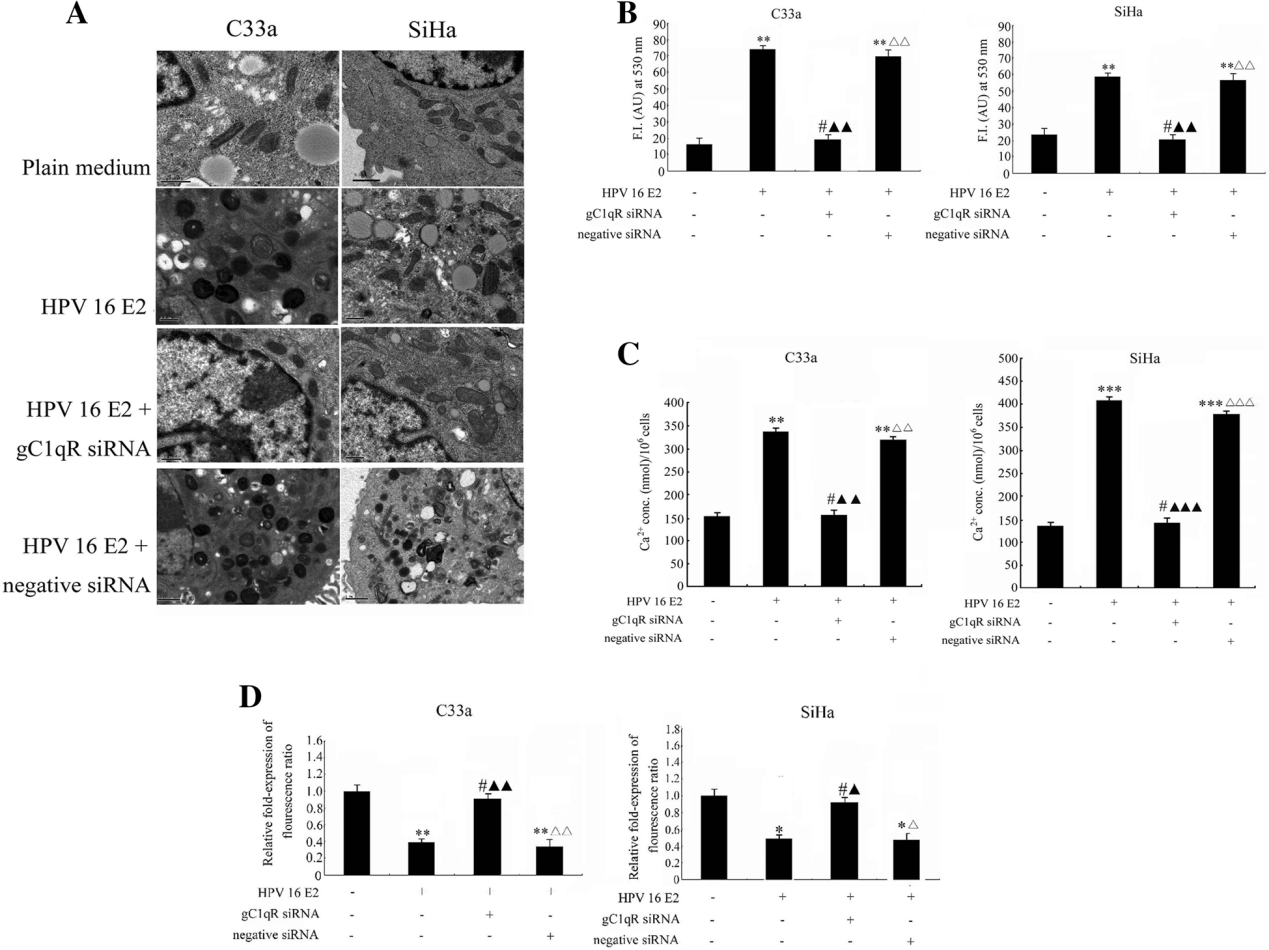
**The effect of gC1qR on HPV-16 E2-induced mitochondrial function. A**: The mitochondrial morphology was observed using electron microscopy. Mitochondrial swelling and vesicular formation were observed in cells transfected with HPV-16 E2 vector or HPV-16 E2 + negative siRNA vector (12500X). **B**: Constitutive expression of HPV-16 E2 following transfection with gC1qR siRNA or negative siRNA induced the production of ROS. ROS generation was quantified by treatment with H_2_DCFDA for 30 min followed by fluorescence microscopy. **p < 0.01, ^#^ *p* > 0.05 versus plain medium group (control); ^▲▲^ *p* < 0.01 versus HPV-16 E2 vector group; ^△△^ *p* < 0.01 versus the HPV-16 E2 + gC1qR siRNA vector group. **C:** Constitutive expression of HPV-16 E2 induced cellular calcium ion influx in the C33a and SiHa cells. **p < 0.01, ***p < 0.001, ^#^ *p* > 0.05 versus plain medium group (control); ^▲▲^ *p* < 0.01, ^▲▲▲^ *p* < 0.001 versus the HPV-16 E2 vector group; ^△△^ *p* < 0.01, ^△△△^ *p* < 0.001 versus the HPV-16 E2 + gC1qR siRNA group. **D**: The mitochondrial membrane potential was measured. The relative Δψm value was detected by monitoring the fluorescence of JC-1 (590: 527 nm fluorescence ratio). **p < 0.01, *p < 0.05, ^#^ *p* > 0.05 versus plain medium group (control); ^▲▲^ *p* < 0.01, ^▲^ *p* < 0.05 versus the HPV-16 E2 vector group; ^△△^ *p* < 0.01, ^△^ *p* < 0.05 versus the HPV-16 E2 + gC1qR siRNA group.

In subsequent experiments, cells were treated with either plain medium, the HPV-16 E2 vector, the HPV-16 E2 + gC1qR siRNA vector, or the HPV-16 E2 + negative siRNA vector for the indicated time periods. High-magnification photomicrographs (12500X) taken with an electron microscope showed severe pathological changes in the mitochondrial morphology (Figure 3A), including mitochondrial swelling and vesicular formation in the C33a and SiHa cells transfected with either the HPV-16 E2 vector or the HPV-16 E2 + negative siRNA vector. In contrast, no obvious changes in the mitochondrial morphology were seen in the cells treated with the plain medium (control) or transfected with the HPV-16 E2 + gC1qR siRNA vector.

Mitochondrial function was assessed by determining changes in ROS generation, cytosolic Ca^2+^ levels, and relative Δψm values. ROS generation was determined using H_2_DCFDA fluorescence and quantified by flow cytometry analysis (Figure 3B). The data showed that ROS levels in the cells treated with either HPV-16 E2 or HPV-16 E2 + negative siRNA vector were significantly increased compared to the control cells. In contrast, a slight change in ROS production was found in the cells treated with HPV-16 E2 + gC1qR siRNA vector compared with plain medium alone (control). The ROS levels were notably decreased in cells transfected with the HPV-16 E2 + gC1qR siRNA vector when compared with cells only transfected with the HPV-16 E2 vector. There is an obvious difference between cells transfected with the HPV-16 E2 + gC1qR siRNA vector and cells transfected with the HPV-16 E2 + negative siRNA vector.

Cytosolic Ca^2+^ levels were determined using a fluorescent ELISA reader (Figure 3C), and the results revealed the [Ca^2+^]i concentration in the HPV-16 E2 vector cells and the HPV- 16 E2 + negative siRNA vector cells was increased compared with the plain medium-treated C33a and SiHa cells. The cells transfected with the HPV-16 E2 + gC1qR siRNA vector showed no changes compared with the plain medium group. However, the [Ca^2+^]i concentration in the HPV-16 E2 + gC1qR siRNA vector cells was notably lower than that in the HPV-16 E2 vector cells. There was an obvious difference between the HPV-16 E2 + gC1qR siRNA vector group and the HPV-16 E2 + negative siRNA vector group after the initial manipulation.

The changes in relative Δψm values in differently treated C33a and SiHa cells was also explored (Figure 3D). We used the JC-1 dye to monitor the estimated Δψm using the 590: 527 nm emission ratio at the specific time points. The Δψm value in the HPV-16 E2 vector group and the HPV-16 E2 + negative siRNA vector group decreased when compared to the plain medium group. There was no difference in the Δψm in the C33a and SiHa cells between the HPV-16 E2 + gC1qR siRNA vector group and plain medium group after the initial manipulation. In contrast, the Δψm values were notably increased in the HPV-16 E2 + gC1qR siRNA vector group compared with the HPV-16 E2 vector alone group. Interestingly, transfection of HPV-16 E2 + negative siRNA vector group significantly decreased the value of Δψm compared with the HPV-16 E2 + gC1qR siRNA vector group.

### The effect of gC1qR on HPV-16 E2-induced apoptosis of C33a and SiHa cells

**Figure 4:**
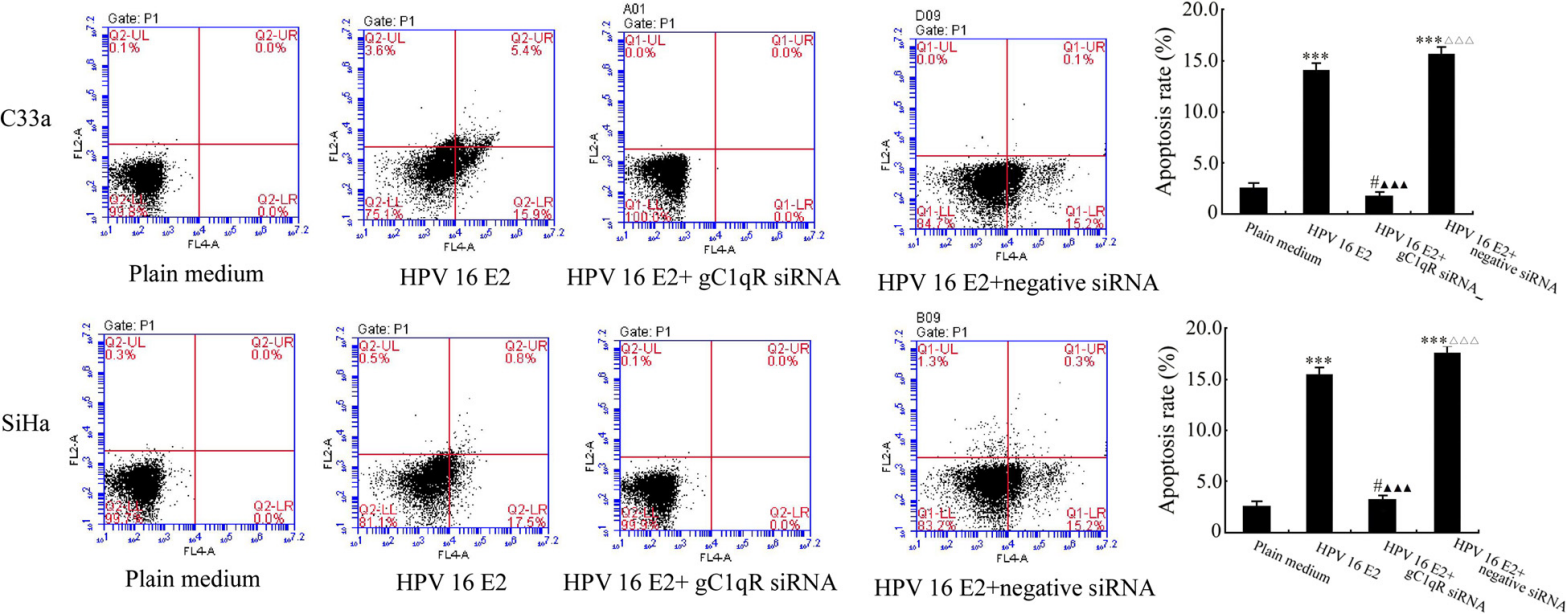
**The effect of gC1qR on HPV-16 E2-induced apoptotic death of cells.** C33a and SiHa cells (2 × 10^6^) were treated with plain medium, HPV-16 E2 vector, HPV-16 E2 vector + gC1qR siRNA vector, or HPV-16 E2 vector + negative siRNA vector. At 48 h post-transfection, the cells were subjected to flow cytometry analysis to detect the levels of apoptosis. The data represent the means ± SD. These data are representative of three independent experiments. ***p < 0.001, ^#^ *p* > 0.05 versus plain medium group (control); ^▲▲▲^ *p* < 0.001 versus the HPV-16 E2 group, ^△△△^ *p* < 0.001 versus the HPV-16 E2 + gC1qR siRNA group.

To evaluate whether silencing the gC1qR gene could reverse HPV-16 E2-induced C33a and SiHa cell apoptosis, cells were treated with either plain medium (control), the HPV-16 E2 vector, the HPV-16 E2 + gC1qR siRNA vector, or the HPV-16 E2 + negative siRNA vector for the indicated time periods. Cells were double-stained with Annexin V and PI (Figure 4). The early and late apoptotic cells were distributed in the Q1_LR and Q1_UR regions, respectively. The necrotic cells were located in the Q1_UL region. Figure 4 shows that cells treated with the HPV-16 E2 vector and the HPV-16 E2 + negative siRNA vector resulted in an increase in the number of cells in the Q1_LR and Q1_UR regions compared with plain medium (control) group. However, there was no difference between the HPV-16 E2 + gC1qR siRNA vector group and the plain medium group (*p* > 0.05). The Q1_LR and Q1_UR regions in the HPV-16 E2 + gC1qR siRNA vector-treated C33a and SiHa cells were diminished compared with the HPV-16 E2 vector group (*p* < 0.001). Treatment of the HPV-16 E2 + negative siRNA vector group significantly increased the apoptotic cells compared with the HPV-16 E2 + gC1qR siRNA vector group.

## Discussion

The results of this study show that over-expression of HPV-16 E2 is associated with apoptosis of human cervical squamous carcinoma cells. Our study has further confirmed that over-expression of HPV-16 E2 could regulate the expression of the gC1qR gene, which induces mitochondrial dysfunction. These findings constitute the first evidence that gC1qR is a target during HPV-16 E2-induced apoptosis in human cervical squamous carcinoma cells.

Experimental evidence has demonstrated that the primary host defence mechanism against viral infection is to target apoptotic proteins, which, when released from the mitochondria, regulate cellular responses including the promotion of cell proliferation or induction of cell death [14]. For example, high-risk HPV-16 E6 was reported to maintain mitochondrial morphology and integrity while inhibiting the release of the pro-apoptotic factor cytochrome c, a potent catalyst of apoptosis [15]. Others studies also demonstrated that apoptosis is triggered by over-expression of HPV-16 E2 by enhancing the expression of pro-apoptotic Bax, inhibiting the expression of anti-apoptotic Bcl xl, releasing cytochrome c from the mitochondria, and activating caspases-9 and -3 [16]. Interestingly, only high-risk HPV E2 proteins, such as the six regulatory molecules (E1, E2, E4, E5, E6, and E7) encoded by the HPV-16 genome, seem to be effective in modulating mitochondrial metabolism [17]. E2 could not only influence the activity of the cell cycle (benign lesions) but may also regulate viral gene expression (including the E6/E7 oncogenes) [18]. Our results demonstrate that E2 transfection into HPV-negative C33A cells or HPV-16-positive SiHa cells increased gC1qR expression, induced mitochondrial dysfunction, and enhanced apoptosis.

The gC1qR protein is primarily localised at the outer mitochondrial membrane [19], enhances ROS production and increases Ca^2+^ uptake by reducing the electron flow from complex I [20]. In recent years, it has become increasingly evident that gC1qR-induced mitochondrial dysfunction is linked to apoptosis and the release of cytotoxic factors such as ROS, which are generated in excess in defective mitochondria. ROS induction, as a by-product, can regulate signalling pathways leading to the inhibition of cell proliferation or cell death [21]. Our previous study demonstrated that gC1qR vector-treated C33a and SiHa cells expressing gC1qR generated increased levels of ROS. Oxidant generation correlated with intracellular Ca^2+^ accumulation and a decrease in the relative Δψm values, which in turn induced cell apoptosis. However, treatment with metformin may reverse gC1qR-induced C33a and SiHa cell apoptosis. This observation was also supported by the results derived from the treatment that silenced the gC1qR gene in HPV-16 E2-induced apoptosis.

## Conclusion

This study has confirmed that E2 upregulates gC1qR gene expression, which induces cervical cancer cell apoptosis. We have also determined that the mechanism by which gC1qR induces apoptosis is through mitochondrial dysfunction in human cervical squamous carcinoma cells. Thus, gC1qR is a key player in the carcinogenesis of HPV-induced cancer and is a potential target for cervical cancer therapy.

## Additional files

Additional file 1: Figure S1. The biological effects of gC1qR over expression on mitochondrial function of C33a and SiHa cells. A: Constitutive expression of gC1qR induced the production of ROS in C33a cells that were transfected with the empty vector or the gC1qR vector for 24, 36, 48, 60, 72, or 84 h. ROS generation was quantified by fluorescence following treatment with H_2_DCFDA for 30 min and then subjected to fluorescence microscopy. The data represent the mean ± SD. These data are representative of three independent experiments. **p* < 0.05, ***p* < 0.01, ****p* < 0.001, ^#^ *p* > 0.05 versus empty vector group. B: Constitutive expression of gC1qR induced cellular calcium ion influx in the C33a and SiHa cells. Quantitative estimation of the intracellular Ca^2+^ levels was monitored using the Fluo-4 AM fluorescence probe in C33a and SiHa cells at different time points ranging from 0 h to 84 h. All of the data are representative of five independent experiments in which the data were calculated by averaging the values as the mean ± SD. **p* < 0.05, ***p* < 0.01, ****p* < 0.001, ^#^ *p* > 0.05 versus empty vector group. C: The mitochondrial membrane potential was observed. Time-dependent changes in the relative Δψm value were observed, as detected by fluorescence of JC-1 (590: 527 nm). These data are representative of three separate experiments. **p* < 0.05, ***p* < 0.01, ****p* < 0.001, ^#^ *p* > 0.05 versus empty vector group. Additional file 2: Figure S2. The effect of HPV-16 E2 on gC1qR expression levels in cervical squamous carcinoma cell lines (C33a and SiHa). Cells were treated with plain medium (control), empty vector, HPV-16 E2 vector or HPV-16 E2 mutant vector for 48 h. A: The relative gC1qR gene expression levels are shown in C33a and SiHa cells. gC1qR expression levels were analysed by real-time PCR. ****p* < 0.001, ***p* < 0.01, ^#^ *p* > 0.05 versus the control group; ^▲▲▲^ *p* < 0.001, ^▲▲^ *p* < 0.01 versus the empty vector group; ^△△△^ *p* < 0.001, ^△△^ *p* < 0.01 versus the HPV-16 E2 group. B: gC1qR protein levels were measured in C33a and SiHa cells using western blot analysis. The graph depicts the relative gC1qR protein levels normalised to actin. The results are expressed as the means ± SD of three separate experiments. ****p* < 0.001, ^#^ *p* > 0.05 versus the control group; ^▲▲▲^ *p* < 0.001 versus the empty vector group; ^△△△^ *p* < 0.001 versus the HPV-16 E2 vector group Additional file 3: Figure S3. The levels of gC1qR expression. C33a and SiHa cells were treated with plain medium (control), negative siRNA, or gC1qR siRNA for 48 h. The expression of the gC1qR protein was measured by western blot analysis. The graph depicts the relative gC1qR protein levels normalised to actin. The results are expressed as the mean ± SD of three separate experiments. **p* < 0.05, ^#^ *p* > 0.05 versus the plain medium group Additional file 4: Figure S4. The effect of HPV-16 E2 on gC1qR expression levels. Cells were treated with plain medium (control) or the HPV-16 E2 vector. After 72 h, the cells were transfected with either 100 ng of the gC1qR siRNA vector or 100 ng of negative siRNA. A: Relative gC1qR gene expression levels in the C33a and SiHa cervical squamous carcinoma cell lines. gC1qR expression levels were analysed by real-time PCR. ****p* < 0.001, ***p* < 0.01, ^#^ *p* > 0.05 versus the HPV-16 E2 (-), gC1qR siRNA (-) and negative siRNA (-) groups; ^▲▲^ *p* < 0.01 versus the HPV-16 E2 (+), gC1qR siRNA (-) and negative siRNA (-) groups group; ^△△^ *p* < 0.01 versus the HPV-16 E2 (+), gC1qR siRNA (+) and negative siRNA (-) groups. B: gC1qR protein levels were measured in C33a and SiHa cells using western blot analysis. The graph depicts the relative gC1qR protein levels normalised to actin. The results are expressed as the means ± SD of three separate experiments. ****p* < 0.001, ***p* < 0.01, ^#^ *p* > 0.05 versus the HPV-16 E2 (-), gC1qR siRNA (-) and negative siRNA (-) groups; ^▲▲▲^ *p* < 0.001, ^▲▲^ *p* < 0.01 versus the HPV-16 E2 (+), gC1qR siRNA (-) and negative siRNA (-) groups; ^△△△^ *p* < 0.001, ^△△^ *p* < 0.01 versus the HPV-16 E2 (+), gC1qR siRNA (+) and negative siRNA (-) groups.

## Abbreviations

HPV-16: Human papillomavirus type 16
gC1qR: Globular heads of C1q receptor
PI: Propidium iodide
cDNA: Complementary DNA
SD: Standard deviation
real-time PCR: Real-time quantitative polymerase chain reaction
