# Supplementary figures and images for: The role of the globular heads of the C1q receptor in HPV-16 E2-induced human cervical squamous carcinoma cell apoptosis via a mitochondria-dependent pathway
Source: J Transl Med. 2014 Oct 5;12:286. doi: 10.1186/s12967-014-0286-y (PMC4194366; doi:10.1186/s12967-014-0286-y)

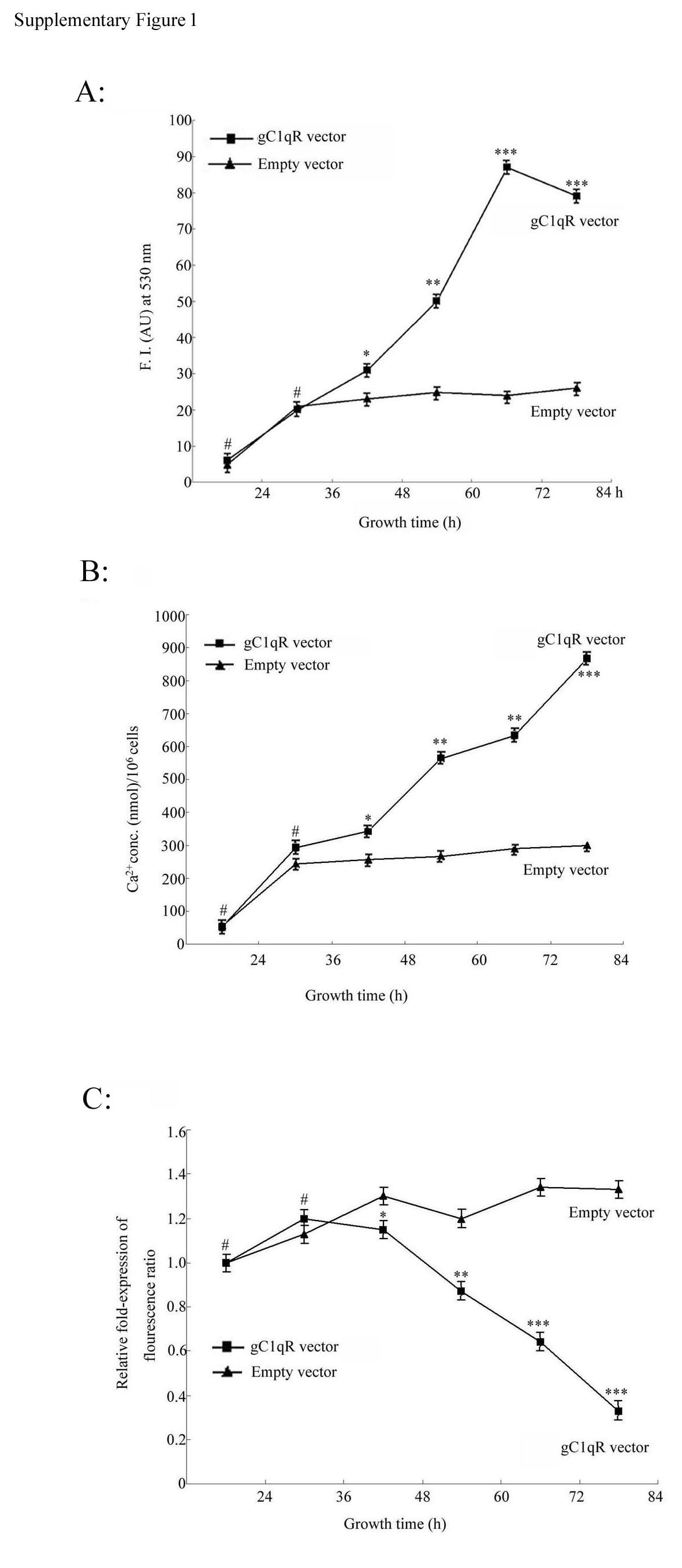

Supplement: Additional file 1: Figure S1. — The biological effects of gC1qR over expression on mitochondrial function of C33a and SiHa cells. A: Constitutive expression of gC1qR induced the production of ROS in C33a cells that were transfected with the empty vector or the gC1qR vector for 24, 36, 48, 60, 72, or 84 h. ROS generation was quantified by fluorescence following treatment with H2DCFDA for 30 min and then subjected to fluorescence microscopy. The data represent the mean ± SD. These data are representative of three independent experiments. *p < 0.05, **p < 0.01, ***p < 0.001, # p > 0.05 versus empty vector group. B: Constitutive expression of gC1qR induced cellular calcium ion influx in the C33a and SiHa cells. Quantitative estimation of the intracellular Ca2+ levels was monitored using the Fluo-4 AM fluorescence probe in C33a and SiHa cells at different time points ranging from 0 h to 84 h. All of the data are representative of five independent experiments in which the data were calculated by averaging the values as the mean ± SD. *p < 0.05, **p < 0.01, ***p < 0.001, # p > 0.05 versus empty vector group. C: The mitochondrial membrane potential was observed. Time-dependent changes in the relative Δψm value were observed, as detected by fluorescence of JC-1 (590: 527 nm). These data are representative of three separate experiments. *p < 0.05, **p < 0.01, ***p < 0.001, # p > 0.05 versus empty vector group. [file 12967_2014_286_MOESM1_ESM.jpeg]

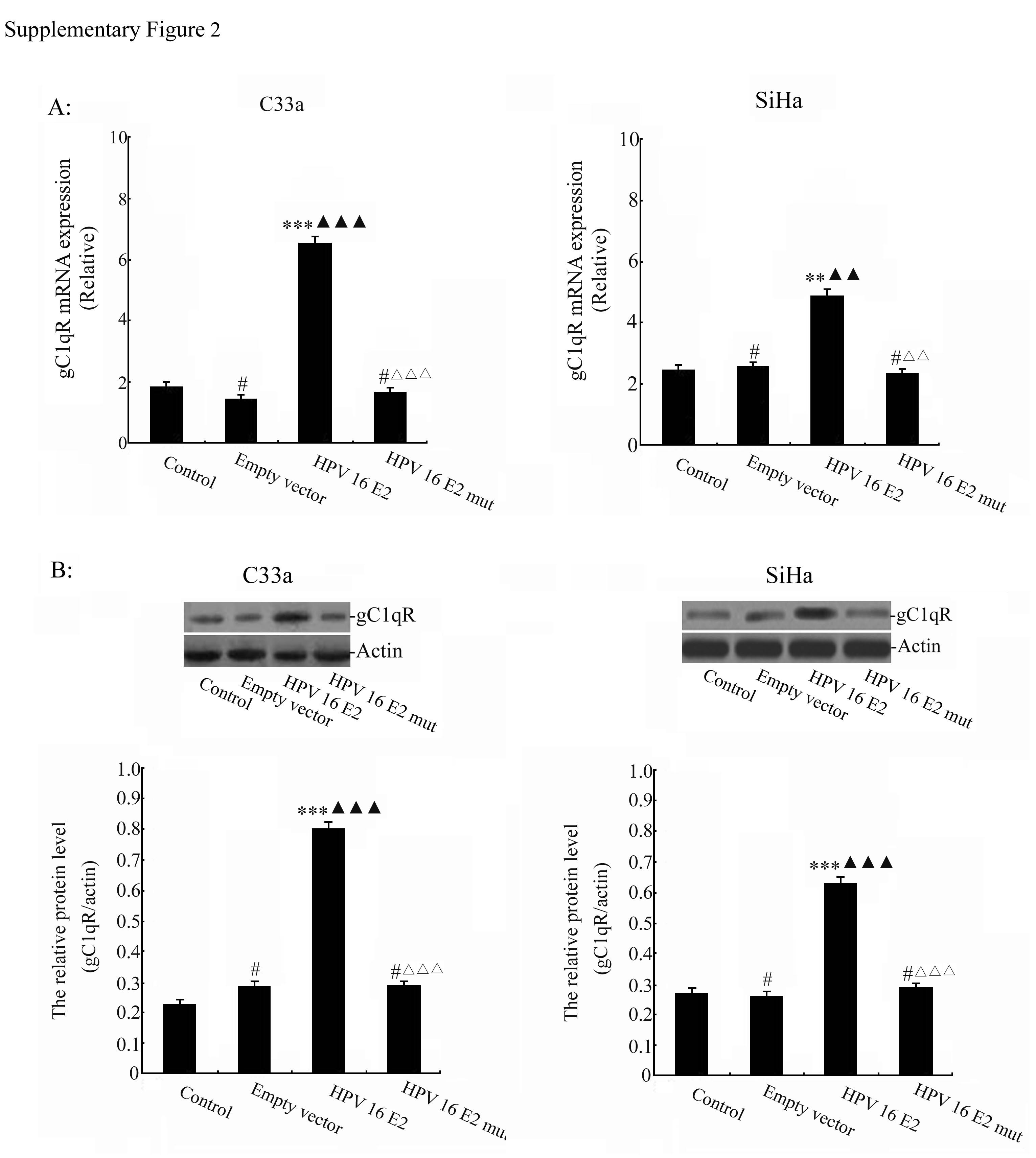

Supplement: Additional file 2: Figure S2. — The effect of HPV-16 E2 on gC1qR expression levels in cervical squamous carcinoma cell lines (C33a and SiHa). Cells were treated with plain medium (control), empty vector, HPV-16 E2 vector or HPV-16 E2 mutant vector for 48 h. A: The relative gC1qR gene expression levels are shown in C33a and SiHa cells. gC1qR expression levels were analysed by real-time PCR. ***p < 0.001, **p < 0.01, # p > 0.05 versus the control group; ▲▲▲ p < 0.001, ▲▲ p < 0.01 versus the empty vector group; △△△ p < 0.001, △△ p < 0.01 versus the HPV-16 E2 group. B: gC1qR protein levels were measured in C33a and SiHa cells using western blot analysis. The graph depicts the relative gC1qR protein levels normalised to actin. The results are expressed as the means ± SD of three separate experiments. ***p < 0.001, # p > 0.05 versus the control group; ▲▲▲ p < 0.001 versus the empty vector group; △△△ p < 0.001 versus the HPV-16 E2 vector group [file 12967_2014_286_MOESM2_ESM.jpeg]

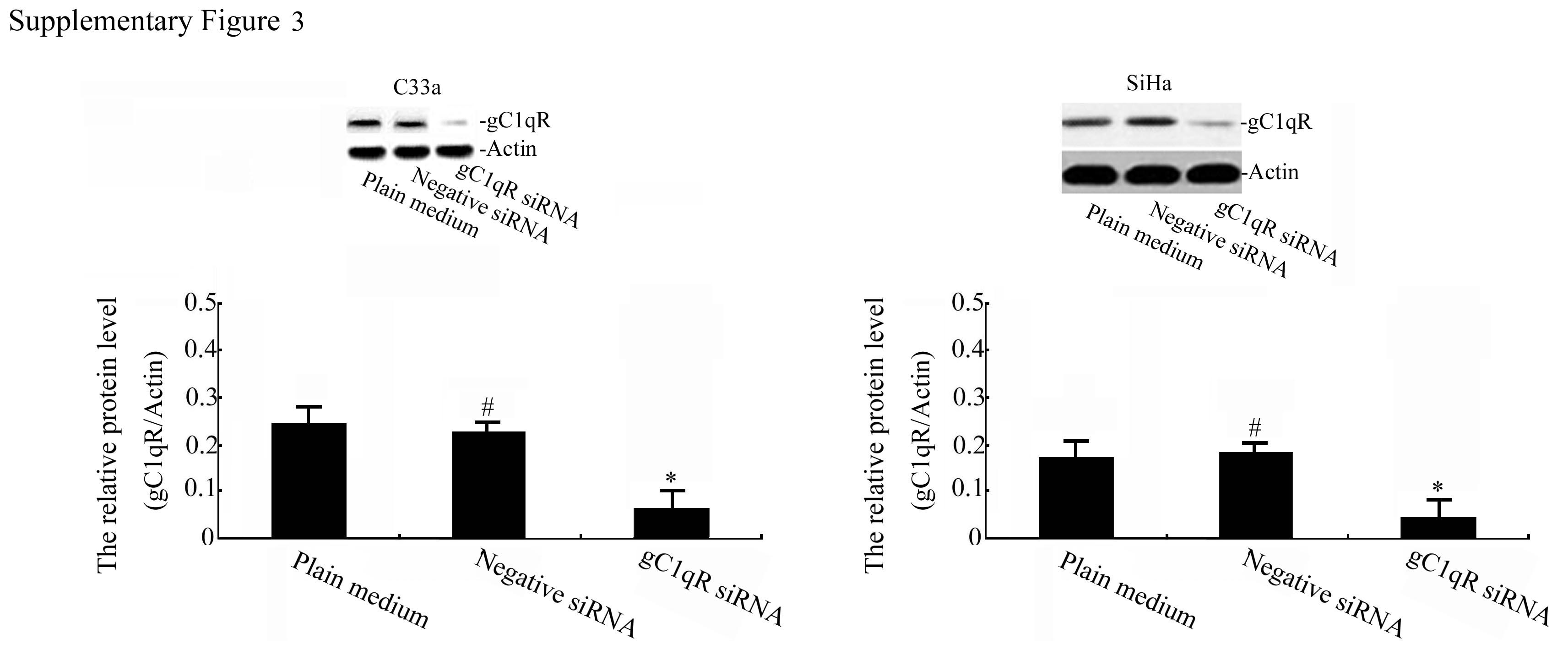

Supplement: Additional file 3: Figure S3. — The levels of gC1qR expression. C33a and SiHa cells were treated with plain medium (control), negative siRNA, or gC1qR siRNA for 48 h. The expression of the gC1qR protein was measured by western blot analysis. The graph depicts the relative gC1qR protein levels normalised to actin. The results are expressed as the mean ± SD of three separate experiments. *p < 0.05, # p > 0.05 versus the plain medium group [file 12967_2014_286_MOESM3_ESM.jpeg]

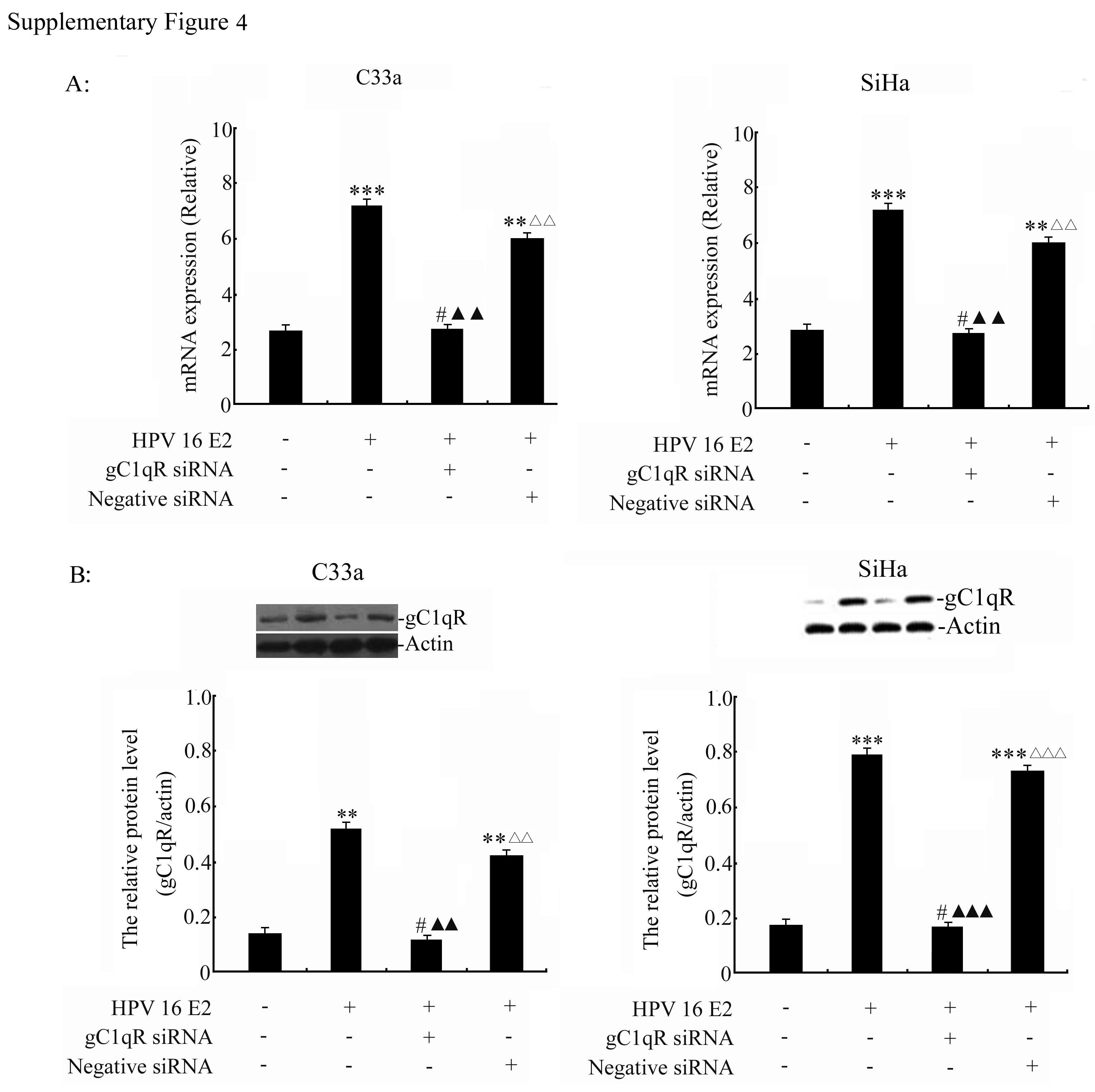

Supplement: Additional file 4: Figure S4. — The effect of HPV-16 E2 on gC1qR expression levels. Cells were treated with plain medium (control) or the HPV-16 E2 vector. After 72 h, the cells were transfected with either 100 ng of the gC1qR siRNA vector or 100 ng of negative siRNA. A: Relative gC1qR gene expression levels in the C33a and SiHa cervical squamous carcinoma cell lines. gC1qR expression levels were analysed by real-time PCR. ***p < 0.001, **p < 0.01, # p > 0.05 versus the HPV-16 E2 (-), gC1qR siRNA (-) and negative siRNA (-) groups; ▲▲ p < 0.01 versus the HPV-16 E2 (+), gC1qR siRNA (-) and negative siRNA (-) groups group; △△ p < 0.01 versus the HPV-16 E2 (+), gC1qR siRNA (+) and negative siRNA (-) groups. B: gC1qR protein levels were measured in C33a and SiHa cells using western blot analysis. The graph depicts the relative gC1qR protein levels normalised to actin. The results are expressed as the means ± SD of three separate experiments. ***p < 0.001, **p < 0.01, # p > 0.05 versus the HPV-16 E2 (-), gC1qR siRNA (-) and negative siRNA (-) groups; ▲▲▲ p < 0.001, ▲▲ p < 0.01 versus the HPV-16 E2 (+), gC1qR siRNA (-) and negative siRNA (-) groups; △△△ p < 0.001, △△ p < 0.01 versus the HPV-16 E2 (+), gC1qR siRNA (+) and negative siRNA (-) groups. [file 12967_2014_286_MOESM4_ESM.jpeg]
